# Prednisone may induce immunologic tolerance by activating the functions of decidual immune cells in early pregnancy

**DOI:** 10.18632/oncotarget.22188

**Published:** 2017-10-31

**Authors:** Xiao-Qian Fu, Jun-Ying Cai, Qian-Yi Huang, Dong-Ju Li, Ning Li, Mu-Jun Li

**Affiliations:** ^1^ Department of Reproductive Center, First Affiliated Hospital of Guangxi Medical University, Guangxi, China; ^2^ Department of Reproductive Center, Maternal and Child Health Hospital and Obstetrics and Gynecology Hospital of Guangxi Zhuang Autonomous Region, Guangxi, China; ^3^ Guangxi Medical University, Guangxi, China

**Keywords:** interleukin-17, prednisone, interleukin-10, Treg cells, Th17 cells

## Abstract

The objective of this study was to investigate alterations in human first-trimester decidual immune cells (DICs) and relevant cytokines after treatment with prednisone. Decidual lymphocytes were treated with prednisone alone, cytokines alone or the combination of prednisone and cytokines. Levels of STAT3, STAT5, RORC and FOXP3 mRNA were assayed using quantitative real-time PCR, proportions of CD4^+^ T helper 17 (Th17) and CD4^+^ T regulatory (Treg) cells were measured using flow cytometry, and concentrations of interleukin (IL)-17A and IL-10 were determined using enzyme-linked immunosorbent assay. After treatment with prednisone alone, levels of STAT5 and FOXP3 mRNA were significantly higher than in untreated control cells (both *P* < 0.01), while levels of RORC mRNA were significantly lower than in controls (*P* < 0.05). Levels of STAT3 mRNA did not vary significantly with treatment. After treatment with prednisone alone, proportions of Th17/CD4^+^ cells and levels of IL-17A were significantly lower than in control cells, and proportions of Treg/CD4^+^ cells and levels of IL-10 significantly higher than in controls (all *P* < 0.01). Our results suggest that prednisone may improve pregnancy outcomes by restoring immunological homeostasis through up-regulation of STAT5 and FOXP3, induction of DIC differentiation into Treg cells, inhibition of DIC differentiation into Th17 cells, reduction of IL-17A secretion and induction of IL-10 secretion.

## INTRODUCTION

Approximately half of patients who suffer at least two pregnancy losses before gestational week 20 are diagnosed with unexplained recurrent spontaneous abortion (URSA) [1], which is largely associated with failure of the maternal-fetal immunological tolerance system [2]. This system plays a critical role in ensuring maternal tolerance of semi-allogeneic fetal antigens, but immune imbalance can induce maternal rejection of the embryo.

T helper 17 (Th17) cells and T regulatory (Treg) cells are CD4^+^ T cells that play opposing roles in maintaining immune balance, and imbalance in their activity may be associated with URSA [3]. Th17 lymphocytes prevent infection, inhibit tumor growth and limit inflammation [4]. Th17 cells differentiated by TGF-β1 and IL-6 or IL-21 express IL-17A, IL-17F, and the “master-regulator” transcription factor RORγt, which is encoded by the RAR-related orphan receptor C (RORC) gene [5]. STAT3 regulates IL-6-induced expression of RORγt as well as IL-17 production [6]. IL-17A is involved in certain inflammatory responses and placental development processes [7]. These processes in Th17 cells drive immune reactions, which when excessive may contribute to URSA. Proportions of Th17 cells in peripheral blood are higher in URSA patients than in healthy pregnant women, suggesting that unbalanced activity by Th17 cells may harm maintenance of pregnancy [8].

Treg cells counterbalance the effects of Th17 cells by keeping CD4^+^ effector T cell responses under control [4]. Many transcription factors, including STAT5 and all-trans retinoic acid receptor, regulate the expression of FOXP3, which when expressed leads Treg cells to inhibit immune responses [9]. This inhibition can impede fetal rejection at the maternal-fetal interface. Creation of a tolerant microenvironment at this interface also involves expression of multiple immuno-modulatory cytokines, such as IL-10 and TGF-β [10]. The endometrial tissue of infertile women, particularly those experiencing repeated failed cycles of *in vitro* fertilization, shows FOXP3 down-regulation [3].

One approach to restoring the balance between Th17 and Treg cell activity and improving pregnancy outcomes is lymphocyte immunization therapy [2]. Another approach is immuno-modulation using the synthetic immunosuppressant prednisone. Prednisone inhibits production and release of histamine and other inflammatory mediators. It also inhibits connective tissue hyperplasia, reduces permeability of capillary walls and cell membranes, and reduces inflammatory exudation. Prednisone can increase the rate of live births by women who have suffered at least three consecutive pregnancy losses [11]. One case report even describes a 35-year-old woman with a history of 13 consecutive spontaneous miscarriages who gave birth after receiving prednisone, aspirin and low-molecular-weight heparin [12]. The success of prednisone therapy for women with recurrent pregnancy loss highlights the need to understand whether and how the drug modulates maternal-fetal immunological tolerance.

Important initial mechanistic insights have come from a study reporting that CD4^+^ T cells differentiate into Th17 cells in the presence of IL-6 and transforming growth factor-β (TGF-β), or into Treg cells in the absence of IL-6 and presence of TGF-β [13]; and from another study implicating IL-23 in maintenance and differentiation of human decidual immune cells (DICs) into Th17 cells [14]. To gain further mechanistic insights, we stimulated DICs with IL-23, IL-6 and TGF-β in order to induce their differentiation into Th17 cells, and then we examined how prednisone affects the balance of Th17 and Treg cells in early pregnancy. Work from our group [14] and others [15] has established this multi-cytokine activation as a reasonable model for simulating pathological alterations in decidual T cells in URSA and analyzing their link to maternal-fetal immunological rejection.

## RESULTS

### Percentages of Th17 and Treg cells in DICs after prednisone treatment

**Figure 1:**
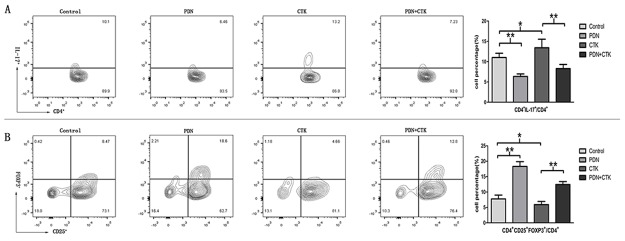
**(A)** Percentages of Th17/CD4^+^ cells in control samples or samples treated with prednisone alone (PDN), cytokines alone (CTK) or the combination of prednisone and cytokines (PDN + CTK). **(B)** Percentages of Treg/CD4^+^ cells in each of the four samples. Results shown are from five independent experiments. ^*^ *P* < 0.05, ^**^ *P* < 0.01.

DICs were treated with prednisone alone, cytokines alone, or the combination of prednisone and cytokines, and then the percentages of DICs that were Th17 (CD4^+^ IL-17^+^)/CD4^+^ or Treg (CD4^+^ CD25^+^FOXP3^+^)/CD4^+^ were compared. The percentages of Th17/CD4^+^ cells were 11.01 ± 1.05% in control cells, 6.35 ± 0.61% in cells treated only with prednisone, 13.42 ± 2.09% in cells treated only with cytokines, and 8.28 ± 1.02% in cells treated with the combination of prednisone and cytokines. Treating cells with cytokines alone led to a significantly higher percentage of Th17/CD4^+^cells than in control cells(*P* < 0.05, Figure 1A). Conversely, treating cells with prednisone alone led to a significantly lower percentage of Th17/CD4^+^ cells than in control cells (*P* < 0.01, Figure 1A). Treating cells with prednisone as well as cytokines led to a significantly lower percentage of Th17/CD4^+^ cells than in cells treated only with cytokines (*P* < 0.01, Figure 1A). This suggests that prednisone inhibits the ability of proinflammatory cytokines to stimulate DIC differentiation into Th17 cells.

Percentages of DIC cells that were Treg/CD4^+^ were 7.76 ± 1.25% in control cells, 18.33 ± 1.54% in cells treated with prednisone alone, 5.93 ± 1.03% in cells treated with cytokines alone, and 12.46 ± 0.95% in cells treated with prednisone and cytokines. Treating cells with cytokines alone led to a significantly lower proportion of Treg cells than in control cells (*P* < 0.05, Figure 1B), while treating them with prednisone led to a significantly higher proportion than in controls (*P* < 0.01, Figure 1B). Treating cells with prednisone as well as cytokines led to a significantly higher percentage of Treg cells than in cells treated with cytokines alone (*P* < 0.01, Figure 1B). This suggests that prednisone weakens the ability of proinflammatory cytokines to inhibit DIC differentiation into Treg cells.

Analysis of the proportions of Th17 and Treg cells suggests that prednisone can influence DIC differentiation into Th17 and Treg cells.

### Effects of prednisone on cytokine production by DICs

**Figure 2:**
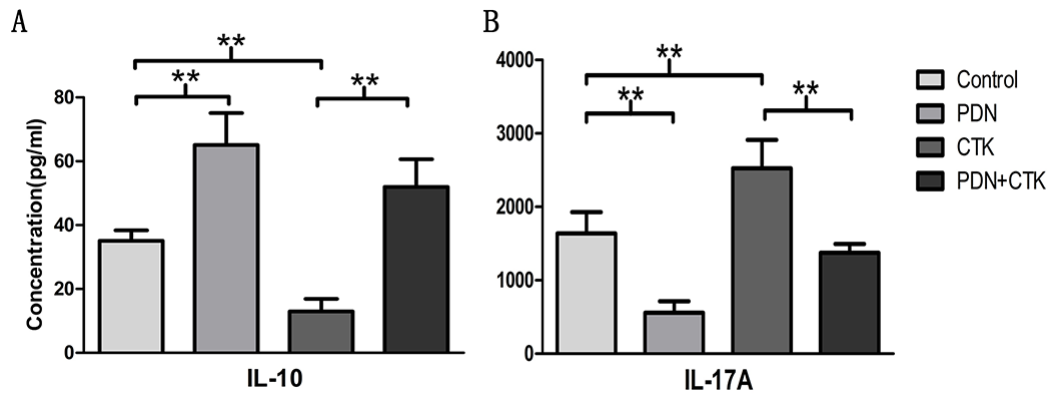
**(A)** IL-10 concentrations by ELISA in supernatants of control cultures and cultures treated with prednisone alone (PDN), cytokines alone (CTK) or the combination of prednisone and cytokines (PDN + CTK). **(B)** IL-17A concentrations by ELISA. Results shown are from six independent experiments. ^**^ *P* < 0.01.

To evaluate Treg cell activity, we assayed culture supernatants for IL-10 following treatment with prednisone alone, cytokines alone, or the combination. (We did not assay for TGF-β, because this was present in the culture medium.) The results mirrored those obtained when we measured the proportion of Treg cells using flow cytometry. IL-10 concentrations (pg/ml) were 35.09 ± 3.25 were in control cells, 65.1± 10.0 in cells treated only with prednisone, 12.96 ± 3.95 in cells treated only with cytokines, and 51.98 ± 8.68 in cells treated with prednisone and cytokines. IL-10 concentration was significantly higher in cells treated with prednisone alone than in control cells (*P* < 0.01, Figure 2A). Inversely, IL-10 concentration was significantly lower in cells treated only with cytokines than in control cells (*P* < 0.01, Figure 2A). IL-10 concentration was significantly higher in cells treated with the combination of prednisone and cytokines than in cells treated only with cytokines (*P* < 0.01, Figure 2A).

To evaluate Th17 cell activity, we assayed culture supernatants for IL-17A after the various treatments. The results were the inverse of those obtained with IL-10. IL-17A concentrations (pg/ml) were 1639.92 ± 289.56 in control cells, 558.79± 155.06 in cells treated with prednisone alone, 2525.47± 385.22 in cells treated with cytokines alone and 1376.12 ± 117.46 in cells treated with the combination of prednisone and cytokines. IL-17A concentration was significantly lower after treatment with prednisone alone than in the control group (*P* < 0.01, Figure 2B), and it was significantly higher after treatment with cytokines alone than in the control group (*P* < 0.01, Figure 2B). IL-17A concentration was significantly lower in cells treated with prednisone and cytokines than in cells treated only with cytokines (*P* < 0.01, Figure 2B).

These results suggest that prednisone can stimulate secretion of anti-inflammatory cytokines such as IL-10. It can inhibit IL-17A secretion, whereas IL-23, IL-6 and TGF-β1 can stimulate IL-17A secretion.

### Levels of RORC, FOXP3, STAT3 and STAT5 mRNAs

**Figure 3:**
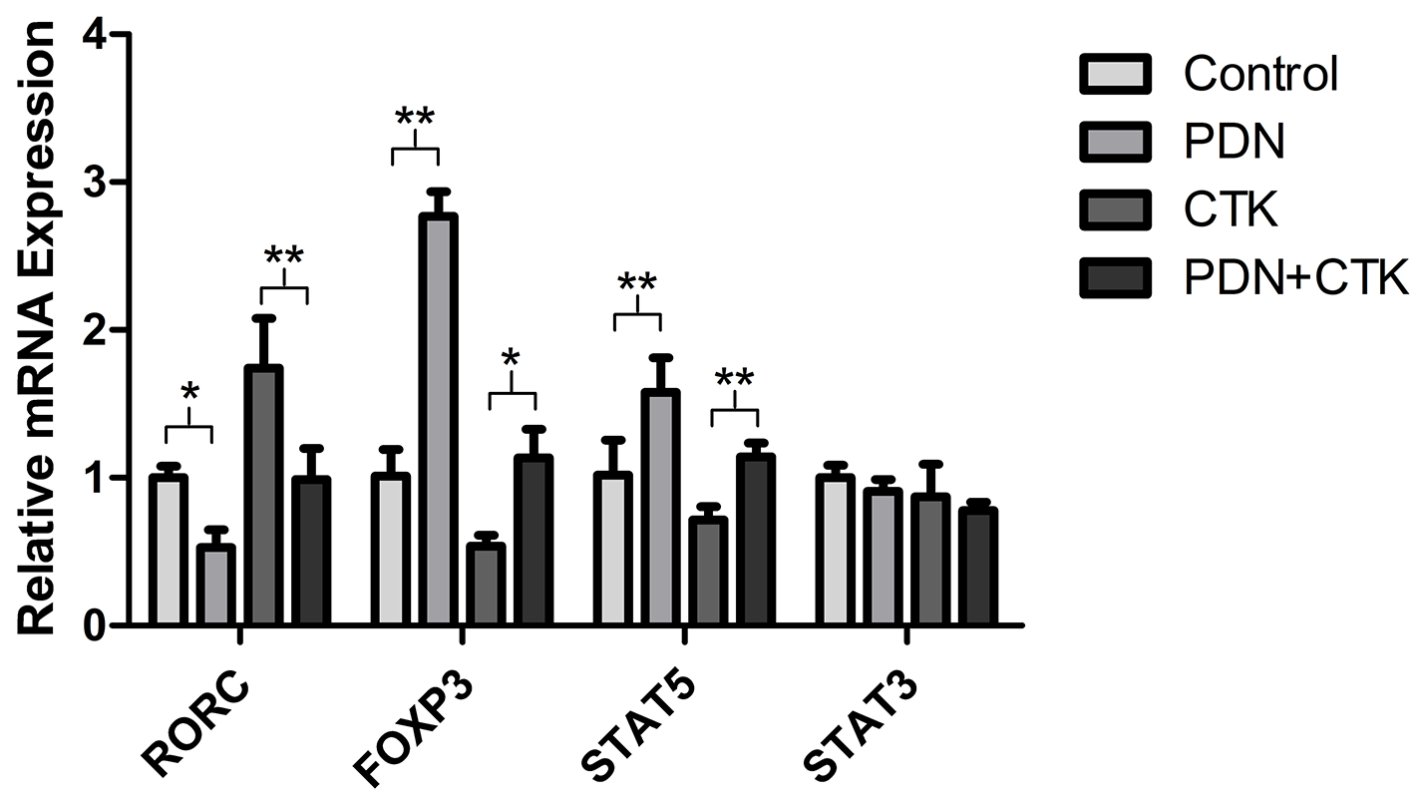
Relative levels of RORC, FOXP3, STAT5 and STAT3 mRNAs in control cultures and cultures treated with prednisone alone (PDN), cytokines alone (CTK) or the combination of prednisone and cytokines (PDN + CTK) Expression levels were normalized to that of GAPDH. Results shown are from three independent experiments. ^*^ *P* <0.05, ^**^ *P* < 0.01.

To evaluate whether prednisone affects RORC transcription, we assayed relative levels of RORC mRNA in cells using quantitative real-time PCR (qRT-PCR) following treatment with prednisone alone, cytokines alone, or the combination. Relative mRNA levels were 1.02 ± 0.08 in control cells, 0.53 ± 0.12 in cells treated only with prednisone, 1.74 ± 0.34 in cells treated only with cytokines and 0.99 ± 0.21 in cells treated with prednisone and cytokines. Levels of RORC mRNA were significantly lower in cells treated with prednisone than in control cells (*P* < 0.01, Figure 3), and they were significantly lower in cells treated with prednisone and cytokines than in cells treated only with cytokines (*P* < 0.05, Figure 3).

We also examined whether prednisone affects relative expression of the transcription factor FOXP3 after the various treatments, since FOXP3 is the most specific functional and phenotypic marker of Treg cells. Based on qRT-PCR, levels of FOXP3 mRNA were approximately 1.7-fold higher in cells treated with prednisone alone than in control cells (*P* < 0.05, Figure 3), and levels were 2.1-fold higher in cells treated with prednisone and cytokines than in cells treated only with cytokines (*P* < 0.01, Figure 3).

Finally, we examined whether prednisone may affect mRNA levels of two STAT transcription factors: STAT3, which is crucial for DIC differentiation into Th17 cells because it regulates T cell proliferation and survival; and STAT5, which activates FOXP3 transcription in Treg cells. All treatment groups and the control group showed similar levels of STAT3 mRNA (all *P* > 0.05, Figure 3), while relative levels of STAT5 mRNA were 1.02 ± 0.24 in control cells, 1.58 ± 0.23 in cells treated only with prednisone, 0.72 ± 0.09 in cells treated only with cytokines and 1.14 ± 0.10 in cells treated with prednisone and cytokines. STAT5 mRNA levels were significantly higher in cells treated with prednisone than in control cells (*P* < 0.01, Figure 3), and they were significantly higher in cells treated with prednisone and cytokines than in cells treated only with cytokines (*P* < 0.01, Figure 3).

These results suggest that prednisone affects transcription of FOXP3, RORC and STAT5, but not STAT3.

## DISCUSSION

Glucocorticoids reduce spontaneous abortions and improve clinical gestation outcomes, and several studies have documented immuno-modulatory effects of prednisone on DICs, but how this synthetic drug acts at the maternal-fetal interface remains to be elucidated. The present study explored the effects of prednisone on lymphocytes in human first-trimester decidua, and it provides evidence that prednisone helps remodel immune balance at the maternal-fetal interface by inducing DIC differentiation into Treg cells, inhibiting DIC differentiation into Th17 cells, reducing IL-17A secretion and increasing IL-10 secretion.

FOXP3^+^ Treg cells mediate immune suppression by inhibiting secretion of proinflammatory cytokines and inhibiting T cells, dendritic cells, and macrophages. This immune suppression requires sustained FOXP3 expression, which is tightly regulated by STAT5 [10]. Glucocorticoid treatment increases the proportion of Treg cells and of related regulatory markers such as GITR, CTLA-4, PD-1, CD73 and FOXP3 [16]. In the present study, treating first-trimester DICs with prednisone up-regulated STAT5, which in turn induced expression of FOXP3 and IL-10. Our experiments suggest that prednisone may inhibit immunological rejection at the maternal-fetal interface by inducing STAT5 expression and thereby stimulating DIC differentiation into Treg cells and IL-10 secretion.

At the same time that prednisone stimulated DIC differentiation into Treg cells, it inhibited DIC differentiation into Th17 cells. One possible mechanism for this inhibition is through down-regulation of STAT3, which initiates differentiation into Th17 cells and which, when phosphorylated, induces expression of RORα and RORγt [6, 17], which in turn trigger secretion of IL-17A, IL-17F and IL-22. However, we failed to detect effects of prednisone on STAT3 transcription; instead, the drug inhibited RORC transcription and IL-17A secretion. We speculate that prednisone indirectly inhibits DIC differentiation into Th17 cells by up-regulating STAT5 and FOXP3. Consistent with this idea, STAT3 and STAT5 share multiple binding sites along the region encoding IL-17, and STAT5 blocks STAT3-driven IL-17 transcription in T cells [18]. In addition, FOXP3 interacts with RORγt to inhibit RORγt-mediated IL-17A transcription [19].

We stimulated the DICs in our study using the combination of plate-bound anti-CD3 antibody, soluble anti-CD28 antibody, and low-dose soluble IL-2 as a polyclonal T cell stimulus independent of antigen-presenting cells. IL-2 can promote Treg survival by binding to its cognate receptor and thereby triggering STAT5 phosphorylation [20].

To simulate alterations in decidual T cells in USRA patients, we added IL-23, IL-6 and TGF-β1 to the culture medium in the present study. The efficacy of this approach was confirmed by our observation that proportions of Th17 cells were higher, and proportions of Treg cells lower, in cells treated only with cytokines than in untreated control cells. Several studies have reported that URSA patients have elevated Th17 cell proportions and reduced Treg cell proportions in peripheral blood [13] and decidua [3, 8]. IL-23 synergizes with IL-6 to induce Th17 cell differentiation, and overexpression of the IL-23 receptor on activated T cells leads to IL-23-dependent differentiation of Th17 cells [15]. TGF-β1 and IL-6 synergistically induce differentiation of Th17 cells [17], and the two factors together induce optimal IL-17 expression by activated T cells. TGF-β1 also induces FOXP3 expression, while IL-6 inhibits its expression [21]. In this way, TGF-β1 regulates Treg and Th17 cells in opposite directions during differentiation.

Our finding that prednisone stimulated DIC differentiation into Treg cells and inhibited DIC differentiation into Th17 cells should translate to improved maternal-fetal immune tolerance. The fact that we observed significant prednisone effects even with strong DIC activation using IL-23, IL-6 and TGF-β1 emphasizes the drug's therapeutic efficacy. At the same time, future work should seek to confirm and extend our findings using other decidual tissue systems activated in different ways. Our research provides testable hypotheses to guide detailed studies into why prednisone can improve pregnancy outcomes for many women. For example, the mechanistic results obtained in the present study may help explain how prednisone can improve pregnancy outcomes in women with unexplained infertility [22] or repeated implantation failure [11]; both conditions have been linked to impaired recruitment of Treg cells or insufficient differentiation of uterine T cells into Treg cells.

Synthetic glucocorticoids such as prednisone are widely prescribed for a variety of inflammatory disorders because of their anti-inflammatory properties. Proper control of inflammation and of immune responses is essential for embryo implantation [23], because it promotes endometrial receptivity, tolerance of the foreign embryo, and vascular adaptation to support placental morphogenesis. Immune responses around the time of conception can also affect fetal growth and gestational age at birth [23]. URSA involves significant inflammation, and this appears to be due to an insufficient number of Treg cells or inadequate activity of available Treg cells, which are then overwhelmed by effector T cells. Efficient control of inflammation is likely essential to promote Treg suppressor function and to remodel the immune balance at the maternal-fetal interface. Glucocorticoids control inflammation by inhibiting cytokine production and proliferation of effector T cells after binding to the glucocorticoid receptor, a member of the nuclear receptor superfamily [24]. This receptor sequesters pro-inflammatory transcription factors such as NF-κB, AP-1, and STATs and prevents their binding to promoters, leading to indirect trans-repression and suppressing cytokine expression [25]. T cells as a whole undergo glucocorticoid-induced apoptosis [26], while IL-2 protects Treg cells from such apoptosis [24]. Cytokines likely inhibit apoptosis by inhibiting glucocorticoid-mediated induction of the NF-κB inhibitor called IĸBα. Glucocorticoids also down-regulate the STAT3 negative regulator called SOCS3, which inhibits IL-6-induced STAT3 phosphorylation. At the same time, glucocorticoids in Th17 cells do not increase IL-6-induced STAT3 phosphorylation [27], so factors other than SOCS3 probably maintain STAT3 activity in these cells.

These considerations highlight the risk that inappropriate corticosteroid administration can impede conception or pregnancy maintenance. Indeed, our experiments showed that prednisone altered the proportions of decidual T cells regardless of whether the immune response was balanced or not. We suggest restricting corticosteroid use to women in whom overt immune pathology has been confirmed. In such women, including those with URSA, unexplained infertility or repeated implantation failure, prednisone and related drugs may improve pregnancy outcomes via their anti-inflammatory and immuno-modulatory effects. Optimal therapy is unlikely to be ‘one-size-fits-all’. More preclinical studies are essential to define patient groups and treatment regimens that show efficacy without inhibiting necessary immune responses. In the present study, we applied the minimum dose that produced significant T cell proliferation in preliminary experiments (data not shown). Future work should carefully optimize doses in animals and consider how those doses translate to humans. Duration of prednisone therapy should also be considered, since long-term treatment may lead to premature membrane rupture and infection.

In conclusion, our data suggest that prednisone may restore immunological homeostasis by inducing STAT5 and FOXP3 expression, inducing DIC differentiation into Treg cells and inhibiting DIC differentiation into Th17 cells. At the same time, prednisone reduces IL-17A secretion and increases IL-10 secretion. Further study should explore DIC immunophenotyping as a tool for investigating immunological disturbances in pregnancy-related disorders such as URSA, unexplained infertility and repeated implantation failure.

## MATERIALS AND METHODS

### Human decidual tissue collection

From May 2016 to August 2016, decidual samples were obtained from 15 healthy pregnant women who sought induced abortions at the Department of Gynecology of the First Affiliated Hospital of Guangxi Medical University. The mean age of the patients was 27.93 ± 3.47 years, and mean gestational age was 53.93 ± 6.96 days. All pregnancies were diagnosed by transvaginal ultrasonography. No patient received treatment such as misoprostol or mifepristone before induced abortion by vacuum aspiration. The Ethics Committee of the First Affiliated Hospital of Guangxi Medical University approved this study, and all participants provided written informed consent for their clinical samples to be used in this work.

Decidual tissues from aborted products were immediately collected in pre-chilled RPMI 1640 medium (Invitrogen, CA, USA) containing 100 IU/mL penicillin and 100 mg/mL streptomycin. Tissues were washed in sterile phosphate-buffered saline (PBS), and then DICs were isolated as described below.

### Isolation and culture of primary DICs

DICs were isolated as described [14]. Briefly, decidual tissues were cut into 1-mm^3^ pieces and digested at 37°C for 1 h with 2 mg/mL collagenase IV (Sigma, St. Louis, MO, USA) and DNase I (Sigma). The cell suspension was successively passed through 100, 300, and 400 mesh screens and centrifuged at 600 *g* in a discontinuous Percoll gradient. DICs ranging in density from 1.056 to 1.077 g/mL were recovered and cultured in 6-well plates in RPMI 1640 supplemented with 10% fetal bovine serum (Gibco, Life Technologies), 100 U/mL penicillin, and 100 mg/mL streptomycin at 37°C in an atmosphere of 5% CO_2_. DICs from different patients were kept separate in light of potential phenotypic differences among subjects.

### Treatment of human first-trimester DICs

DICs were seeded into 6-well plates (2.0 × 10^6^/well) pretreated overnight at 4°C with 10 μg/mL purified anti-CD3 antibody (eBioscience, San Diego, CA, USA). Then DICs were stimulated by adding recombinant human IL-2 (50 ng/ml; Peprotech, Rocky Hill, NJ, USA) and anti-CD28 antibodies (1 mg/ml; eBioscience) to the medium. Stimulated cultures were then divided into four groups, which were further treated for 72 h with nothing (control); with 1 μM prednisone alone (PDN); with 10 ng/ml IL-23, 20 ng/ml IL-6, and 5 ng/ml TGF-β1 as proinflammatory cytokines (CTK); or the three proinflammatory cytokines for 24 h, followed by 1 μM prednisone for 48 h (PDN+CTK). Each treatment was performed in triplicate.

### Quantitative real-time PCR

After cultures were treated as described above, 2 × 10^6^ DICs were collected. Total RNA was extracted using TRIzol reagent (Invitrogen, CA, USA), and cDNA was generated using 5X All-In-One RT MasterMix (Applied Biological Materials, Canada) according to the manufacturer's instructions. Quantitative real-time PCR was performed on the Agilent Strata Gene Mx3005 (Stratagene, USA) using primers specific for human STAT3 (primer ID HQP017767), human STAT5 (HQP017774), human FOXP3 (HQP055129) or human RORC (HQP016379). Results were expressed relative to expression of glyceraldehyde-3-phosphatedehydrogenase (primer ID HQP006940) using the 2^-Δ Δ Ct^ cycle threshold method. All primers were synthesized and their specificity verified by GeneCopoeia (Rockville, MD, USA). Samples underwent 40 cycles of amplification in a volume of 20 μl using the EvaGreen 5X qPCR MasterMix (Applied Biological Materials). Amplification conditions were 95°C for 10 min, followed by 40 cycles of 15 s at 95°C and 60 s at 60°C. Fluorescence data were acquired at 72-95°C in order to minimize nonspecific signal. Specificity of amplification was confirmed by calculating melting curve profiles at the end of each PCR. Each sample was analyzed in triplicate.

### Flow cytometry

In the IL-17 assay, cells were stimulated for 5 h with phorbol 12-myristate 13-acetate (25 ng/ml), ionomycin (1 μg/ml) and Golgistop (2 μl, BD Pharmingen, San Jose, CA, USA). Then they were stained with PerCP/Cy 5.5-conjugated anti-CD4 antibody for 30 min at 4°C in the dark, washed with PBS, resuspended in Fix/Perm buffer (BD Pharmingen), and incubated for 30 min at 4°C in the dark. Cells were washed twice with Perm buffer, then stained intracellularly for 30 min with phycoerythrin (PE)-conjugated anti-human IL-17 antibody (BD Pharmingen). To analyze Treg cells, DICs were stained with the following antibodies (BD Pharmingen): PerCP-Cy5.5- conjugated anti-CD4, PE-conjugated anti-CD25, and Alexa Fluor 647-conjugated anti-FOXP3.

**Figure 4:**
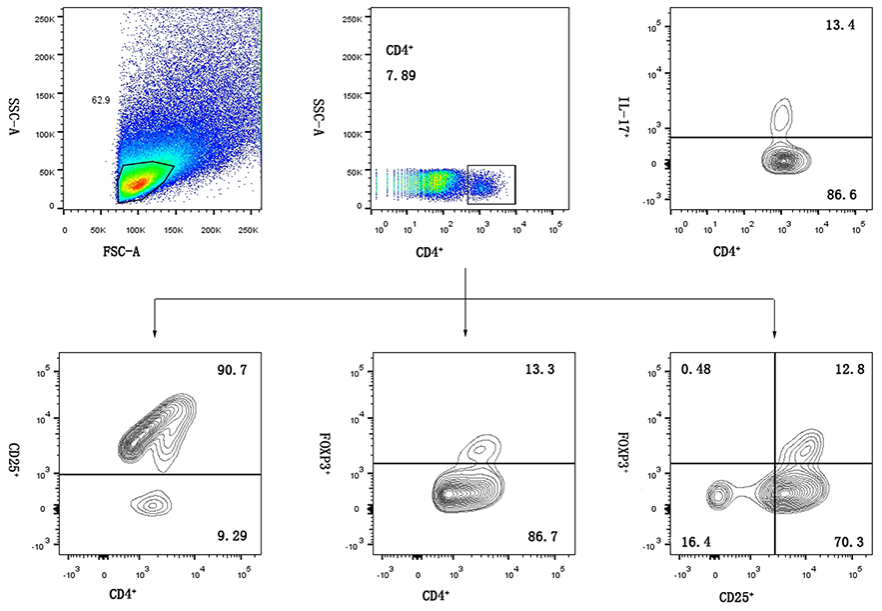
Representative flow chart of decidual lymphocyte immunophenotyping using a sequential gating method Initial gating was performed on forward *vs.* side scatter (FSC/SSC) to identify lymphocytes, then CD4^+^ cells were gated on lymphocytes and separately used to count cells.

Stained cells were processed on a BD flow cytometer equipped with CellQuest software and analyzed using FlowJo 10 software (Treestar, Ashland, OR, USA). Figure 4 shows representative flow cytometry dot plots illustrating the method for detecting and classifying cell subpopulations.

### Enzyme-linked immunosorbent assay (ELISA)

After cultures were treated as described in “Treatment of human first-trimester DICs”, culture medium was collected and centrifuged at 450 *g* for 5 min. The supernatant was assayed for IL-10 and IL-17A using commercial ELISA kits according to the manufacturer's instructions (RayBiotech, GA, USA). These kits had a sensitivity of >1 pg/ml against IL-10 and >80 pg/ml against IL-17A. All samples were measured in triplicate.

### Statistical analysis

Data are presented as mean ± standard deviation. Statistical analysis of differences between two groups was performed using the two-sided Student's *t* test, while differences among more than two groups were analyzed using one-way ANOVA. All statistical analyses were performed using SPSS 17.0 (IBM, Chicago, IL, USA). *P* < 0.05 was considered statistically significant.
